# Perilla-Leaf-Derived Extracellular Vesicles Selectively Inhibit Breast Cancer Cell Proliferation and Invasion

**DOI:** 10.3390/ijms242115633

**Published:** 2023-10-26

**Authors:** Do Kyung Kim, Su Jin Kang, Won Jong Rhee

**Affiliations:** 1 Department of Bioengineering and Nano-Bioengineering, Incheon National University, Incheon 22012, Republic of Korea; ehrud2114@gmail.com (D.K.K.); sjkang2525@gmail.com (S.J.K.); 2 Division of Bioengineering, Incheon National University, Incheon 22012, Republic of Korea; 3 Research Center for Bio Materials & Process Development, Incheon National University, 119 Academy-ro, Yeonsu-gu, Incheon 22012, Republic of Korea

**Keywords:** breast cancer, extracellular vesicle, perilla leaf, Perex, caveolin-1

## Abstract

Breast cancer is a common type of cancer characterized by high mortality rates. However, chemotherapy is not selective and often leads to side-effects. Therefore, there is a need for the development of highly efficient drugs. Recent studies have shown that some extracellular vesicles (EVs) derived from cell cultures possess anti-cancer activity and hold great potential as cancer therapeutics. However, the use of mammalian cell cultures for EV production results in low productivity and high costs. To address this issue, extracellular vesicles derived from perilla leaves (Perex) were isolated and investigated for their anti-cancer activity in various cancer cells. Initially, a high concentration of Perex with a low level of impurities was successfully purified through a combination of ultrafiltration and size-exclusion chromatography. Perex exhibited potent anti-cancer activities, inhibiting the proliferation, migration, and invasion of MDA-MB-231 cancer cells, which have high levels of caveolin-1 compared to other cancer and normal cells. This selective attack on cancer cells with high levels of caveolin-1 reduces unwanted side-effects on normal cells. Considering its high productivity, low production cost, selective anti-cancer activity, and minimal side-effects, Perex represents a promising candidate for the therapeutic treatment of breast cancer.

## 1. Introduction

Breast cancer is the most common cancer and second leading cause of death among women worldwide [1,2]. Despite recent improvements in survival rates, metastatic cancer is considered an incurable disease, and breast cancer mostly metastasizes to the lungs, liver, and bone [3,4]. Therefore, inhibiting the proliferation and metastasis of breast cancer cells is important for improving their survival rate and therapeutic efficiency. Human breast cancers are classified based on endocrine gene expression, including the expression of the estrogen receptor (ER), human epidermal growth receptor 2 (HER2), and progesterone receptor (PR). MCF-7 and MDA-MB-231 are the most commonly used human breast cancer cell lines. MCF-7 cells are positive for both the estrogen receptor (ER) and progesterone receptor (PR), whereas MDA-MB-231 cells are classified as triple-negative breast cancer (TNBC). Triple-negative breast cancer is generally the most aggressive and invasive, with a poorer survival prognosis due to the absence of these three receptors, making it insensitive to anti-estrogen drugs. Therefore, there is a need to develop drugs for the treatment of TNBC [5,6,7]. However, traditional chemotherapy for breast cancer has limitations including its cytotoxicity to normal cells, poor biocompatibility, uncontrolled drug release, and drug resistance [8,9,10]. Nanomedicine has received significant attention to overcome the limitations of cancer pharmacotherapy and has been extensively studied over the past several decades.

Extracellular vesicles (EVs) are small lipid-bound vesicles surrounded by a phospholipid bilayer produced by most cell types and secreted into the extracellular environment [11,12,13,14]. EVs participate in cell-to-cell communication by transferring various biomolecules, including DNA, RNA, proteins, and lipids [15,16]. In recent years, mammalian-cell-derived EVs have been extensively studied as novel biomaterials for biomarker sources, cell therapy surrogates, and drug delivery vehicles [17,18,19,20,21,22]. For instance, mesenchymal-stem-cell-derived EVs have shown therapeutic potency in angiogenesis, tissue regeneration, and immune regulation [23,24]. However, the clinical application of mammalian-cell-culture-derived EVs for therapeutic purposes is limited because of their low yields. Therefore, EV production at a large scale is required to procure the amount of EVs required for human clinical trials. In addition, the use of animal-derived components, including fetal bovine serum (FBS), should be avoided during the production of mammalian-cell-derived EVs because of safety issues associated with drug approval [25,26]. Therefore, it is necessary to investigate alternative sources of EVs to overcome these limitations. 

Recently, plant-derived EVs have shown great potential as alternative sources of mammalian-cell-derived EVs. Plant-derived EVs have biophysical properties similar to those of mammalian-cell-derived EVs in terms of shape, size, and therapeutic activities [27,28,29,30,31]. In particular, plant-derived EVs have been recently isolated from commonly cultivated foods throughout the world, including garlic, cabbage, ginger, and broccoli [32,33,34,35]. These EVs have potential therapeutic effects, such as anti-inflammatory [33,36,37], anti-oxidative [38,39], and anti-cancer activities [40,41,42]. However, few studies have investigated the efficient isolation and characterization of plant-derived EVs. Moreover, most plant-derived EVs show unwanted side-effects by affecting normal cells in addition to cancer cells. Thus, the effect of plant-derived EVs on cancer cell proliferation and metastasis needs to be investigated. 

**Figure 1:**
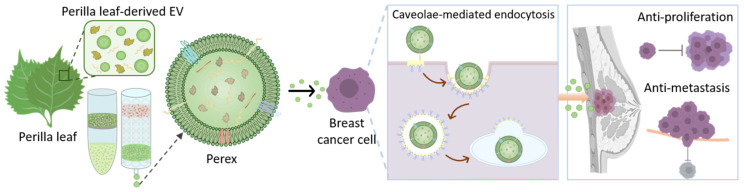
Schematic illustration of perilla-leaf-derived extracellular vesicle (Perex) isolation from perilla leaves and the investigation of its anti-cancer effects and endocytosis mechanism in breast cancer cells.

In this study, perilla (*Perilla frutescens*) leaves were used as a novel source of plant-derived EVs, because they are easily and widely cultivated. Perilla-derived EVs (Perex) were investigated for their anti-cancer effects on normal and cancer cells. Notably, we found that Perex exerted an anti-proliferative effect on specific breast cancer cells without affecting other cancer and normal cells. Perex exerts an anti-metastatic effect by suppressing the migration and invasion of breast cancer cells (Figure 1). The selective effect of Perex on breast cancer cells was also elucidated. Considering its strong anti-cancer effect, high productivity, and low toxicity to normal cells, Perex holds great potential as a novel biomaterial and provides a new strategy to treat cancer, including breast cancer. 

## 2. Results and Discussion

### 2.1. Isolation of Perex from Perilla Leaves Using Ultrafiltration and Size-Exclusion Chromatography

**Figure 2:**
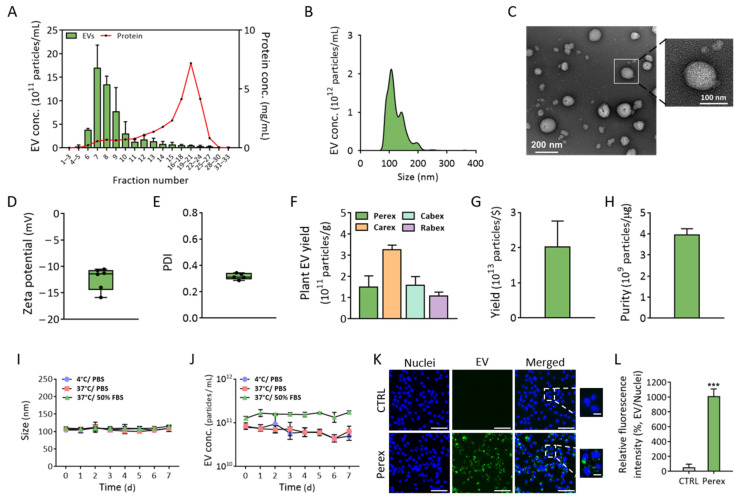
Isolation and characterization of perilla-leaf-derived extracellular vesicles. (**A**) EVs were isolated from perilla leaves using ultrafiltration to concentrate them followed by purification using size-exclusion chromatography. The concentration of EVs and proteins in all fractions was analyzed. The EVs in fractions 7 and 8 were named Perex and used for the following experiments: (**B**) size distribution of Perex was analyzed using NTA; (**C**) morphology of Perex was observed using TEM; (**D**) zeta potential and (**E**) PDI of Perex were measured using DLS; (**F**) plant-derived EV yield per 1 g of the plant biomass; (**G**) yield per cost for perilla leaves; (**H**) purity per μg of protein impurity; (**I**,**J**) stability of Perex over time in terms of size and EV concentration under different temperatures and environmental conditions; (**K**) uptake of PKH67-dye-labeled Perex delivered to MDA-MB-231 cells. The enlarged images are shown in dashed boxes (the size bars indicate 100 μm; enlarged image scale bar: 20 μm); (**L**) the relative fluorescence intensity of Perex uptaken by cells. All values are expressed as mean ± SD (*** *p* < 0.001; *n* = 3).

EVs should be isolated from perilla leaves with a high yield and purity to investigate the anti-cancer activities of Perex. Ultracentrifugation is the widely used method for the isolation of EVs; however, it can be time-consuming and cause a disruption of EVs due to its high forces, which may render EVs unsuitable as therapeutic agents. In addition, polyethylene-glycol-based precipitation methods involve the coprecipitation of large amounts of impurities [43,44]. In a previous study, EVs from cabbage and carrot were successfully isolated using ultrafiltration followed by size-exclusion chromatography for purification [33,39]. Each eluted fraction was analyzed for EV size, concentration, and protein impurities using nanoparticle tracking analysis (NTA) and bicinchoninic acid (BCA) assays (33 fractions total). A high concentration of EVs was collected in fractions 7 and 8, whereas most protein impurities were eluted and collected in fractions 15 to 27, indicating that EVs were successfully separated from protein impurities from perilla leaves (Figure 2A). The EVs in fractions 7 and 8 were collected, denoted as Perex, and further characterized.

### 2.2. Characterization of Perilla-Leaf-Derived EVs

The biophysical properties of Perex were characterized after isolation using size-exclusion chromatography. NTA showed that the average size of Perex was 118.2 nm, which was within the range of known sizes of EVs (Figure 2B). The morphology of Perex was investigated using transmission electron microscopy (TEM), and the results showed that Perex retained spherical shapes with an average size of approximately 100 nm (Figure 2C). The zeta potential and PDI of Perex were further analyzed using dynamic light scattering (DLS) (Figure 2D,E). The average zeta potential was −12.3 mV, indicating that the membrane of Perex was negatively charged probably due to negatively charged lipids, and the PDI value was 0.36.

The production yield and biological activity of EVs are important for their utilization for therapeutic purposes. EV production using cell cultures requires a large production scale, which is accompanied by high production costs and long culture times. In this context, the production yield based on the weight and price of perilla leaves was assessed to demonstrate the advantage of developing Perex as a novel anticancer therapeutic drug. First, it is noteworthy that the amount of Perex produced from only 1 g of perilla leaves was 1.48 × 10^11^ particles/g, indicating that a high amount of Perex can be produced even with a small amount of perilla leaves (Figure 2F). We also isolated EVs from other plants, including cabbage (Cabex), carrot (Carex), and red cabbage (Rabex), to compare the EV productivity per gram. Perex productivity was as high as those of other plant EVs, and they were 3.24 × 10^11^, 1.57 × 10^11^, and 1.1 × 10^11^ particles/g for Cabex, Carex, and Rabex, respectively. Considering that the average price of perilla leaves is low (0.0074 USD/1 g of perilla leaves), the Perex production yield (1.5 × 10^11^ particles/1 g of perilla leaves) per US dollar was 2.02 × 10^13^ particles/USD (Figure 2G). More than USD 200,000 is required to produce 5 × 10^14^ particles (2.50 × 10^9^ particles/USD) of EVs using human mesenchymal stem cells [45]. Therefore, the extremely high yield and low cost of Perex production are beneficial for its development as a novel therapeutic agent. The purity of Perex (particles/μg of protein) was 4.00 × 10^9^ particles/μg calculated by dividing the Perex particle concentration by the protein concentrations in Perex fractions (fractions 7 and 8) (Figure 2H). Considering that the purity of isolated EVs produced from human cells using the same EV purification processes (ultrafiltration followed by size-exclusion chromatography) was 0.75 × 10^9^ particles/μg [46], Perex had fewer protein impurities.

### 2.3. Perex Stability and Cellular Internalization of Perex in Breast Cancer Cells

The stability of Perex at 4 °C or 37 °C in phosphate-buffered saline (PBS) and 50% serum conditions was assessed by analyzing the size and concentration changes to determine its prospects for clinical applications (Figure 2I,J). Perex exhibited no significant changes in size for 7 days, regardless of the temperature and environment (Figure 2I). There was a slight decrease in the Perex concentration when the incubation time increased, indicating that there was some Perex loss (Figure 2J). However, the Perex concentration was relatively constant under physiological conditions, even when Perex was incubated for 7 days with serum. 

The uptake of Perex by mammalian cells was also investigated. EVs participate in cell-to-cell communication by binding to or entering the cells. Thus, we presumed that Perex is also internalized into cells and transfers biomolecules to cells to exert biological effects. Perex was stained with PKH67 dye followed by supplementation of the cell culture medium after the elimination of unstained dye to demonstrate its cellular uptake. MDA-MB-231 cells were cultured in a medium supplemented with 1.0 × 10^11^ particles/mL of PKH-67-labeled Perex at 37 °C for 6 h, and the nuclei were stained with Hoechst 33342. A high fluorescence was detected in Perex-treated MDA-MB-231 cells, indicating that Perex was internalized by the mammalian cells (Figure 2K). The uptake efficiency of penetrated Perex was approximately 19-fold higher compared to the CTRL group (Figure 2L). Therefore, we can conclude that Perex can deliver biomolecules to mammalian cells, which may subsequently regulate the biological activities of target cells.

### 2.4. Cytotoxic Effects of Perex against Various Cell Types

**Figure 3:**
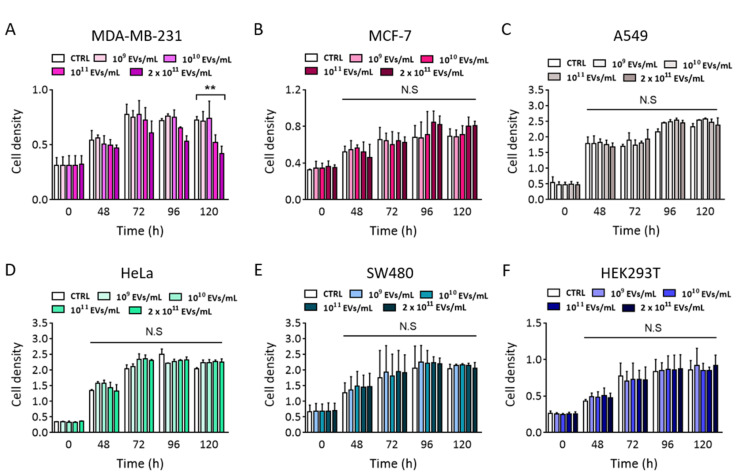
Cytotoxic effects of Perex on different cell types, including cancer and normal cells. (**A**) MDA-MB-231 (human breast cancer), (**B**) MCF-7 (human breast carcinoma), (**C**) A549 (human lung cancer), (**D**) HeLa (human cervical cancer), (**E**) SW480 (human colorectal cancer), and (**F**) HEK293T (human embryonic kidney) were supplemented with different concentrations of Perex, and cell proliferation rates were measured at 0, 48, 72, 96, and 120 h using the WST-1 assay. All values are expressed as mean ± SD (** *p* < 0.01, N.S: not significant; *n* = 3).

The cytotoxic effects of Perex were observed in five representative cancer cell types, including MDA-MB-231, MCF-7, A549, HeLa, and SW480. Non-cancerous normal cells, including HEK293T, were also tested for Perex cytotoxicity. All cells were supplemented with various concentrations of Perex, ranging from 1 × 10^9^ to 2 × 10^11^ particles/mL, in the culture medium. Notably, time- and concentration-dependent cytotoxic effects of Perex were observed only in MDA-MB-231 cells (Figure 3A). For instance, 28% and 42% decreases in cell density were observed when MDA-MB-231 cells were supplemented with 1 × 10^11^ and 2 × 10^11^ particles/mL Perex for 120 h, respectively. In contrast, no significant decrease in cell density was observed for the other cancer cells (Figure 3B–E). In addition, no cytotoxicity was observed in the normal cell line, even with a high Perex concentration (Figure 3F). These results demonstrate the selective anticancer effect of Perex on MDA-MB-231 breast cancer cells with relatively low cytotoxicity in normal cells.

### 2.5. Effect of Perex on MDA-MB-231 Cell Migration and Invasion

**Figure 4:**
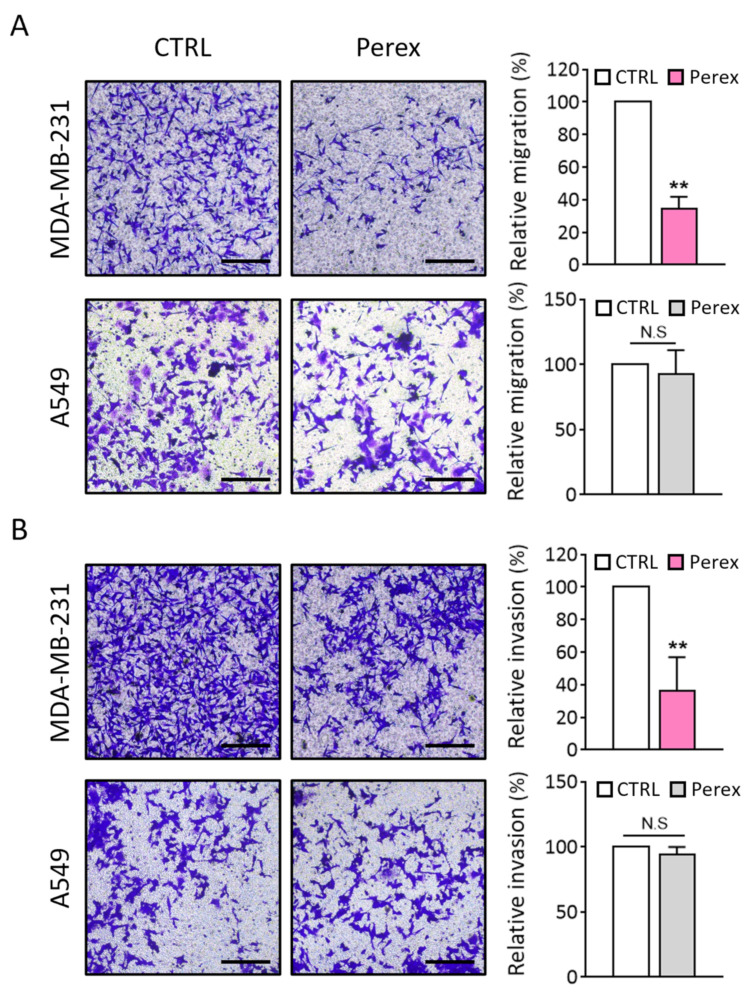
Anti-metastatic effect of Perex on the MDA-MB-231 cells. Cells were supplemented with 2 × 10^11^ particles/mL of Perex for 48 h. A549 cells were used as control. (**A**) Migration assay was performed to determine the effect of Perex on MDA-MB-231 (upper) and A549 (lower) cells. The quantitative analysis of the relative migration capacity by measuring the number of migrated cells is shown on the right. (**B**) Invasion assay was performed to determine the effect of Perex on the invasion of MDA-MB-231 (upper) and A549 (lower) cells. The quantitative analysis of the relative invasion capacity by measuring the number of invaded cells is shown on the right. The size bars indicate 100 μm. All values are expressed as mean ± SD (** *p* < 0.01, N.S: not significant; *n* = 3).

Metastasis is the leading cause of death in patients with breast cancer, and cancer cell metastasis is closely related to its migration and invasion ability. MDA-MB-231 is an aggressive metastatic cancer cell line that exhibits high migration and invasion capabilities. For effective cancer treatment, the migration and invasion activities of breast cancer cells should be inhibited. As Perex selectively inhibited the proliferation of MDA-MB-231 cells, the effect of Perex on MDA-MB-231 cell migration was explored using a Transwell assay (Figure 4). A549 lung cancer cells were also tested for comparison because Perex did not affect A549 cell proliferation (Figure 3C). Both cell lines were supplemented with 2 × 10^11^ particles/mL of Perex for 48 h. Perex showed a significant inhibitory effect on cell migration in MDA-MB-231 cells, with a 65.8% decrease in cell migration compared to the unsupplemented control (Figure 4A). However, no significant decrease in cell migration was observed when A549 cells were treated with Perex. 

To evaluate the anti-metastatic effect of Perex, cell invasion assays for MDA-MB-231 and A549 cells were performed using Transwell inserts coated with 200 μg/mL of Matrigel. The number of invasive cells drastically decreased to 36.5% in MDA-MB-231 cells treated with 2 × 10^11^ particles/mL of Perex compared to the untreated control (Figure 4B). Again, Perex supplementation did not reduce the number of invasive A549 cells. Thus, Perex suppressed the migration and invasion of MDA-MB-231 cells, thereby inhibiting the capability of malignant breast cancer metastasis, while no effect was observed in other cancer cell types. 

### 2.6. Investigation of Selective Anti-Cancer Effects of Perex on MDA-MB-231 Cells

**Figure 5:**
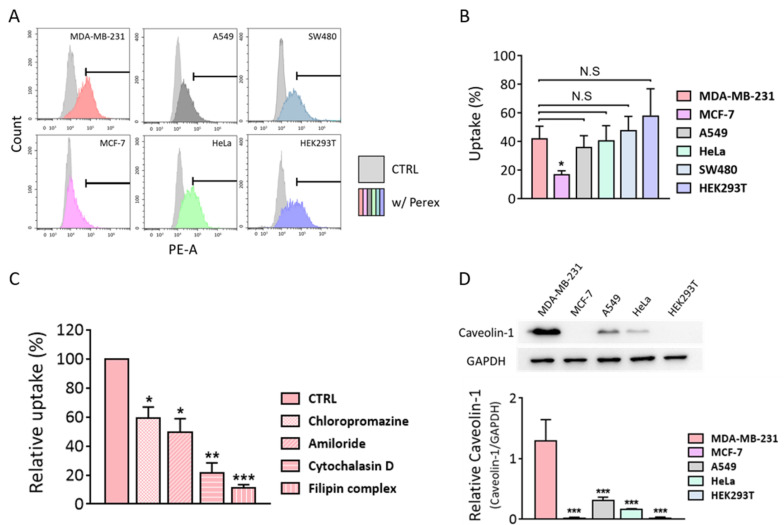
Investigation of Perex uptake mechanism. (**A**,**B**) DiI-labeled Perex was delivered to various cell types and the uptake rates were measured using flow cytometry. (**C**) Cellular uptake of Perex by MDA-MB-231 cells pre-treated with chlorpromazine (clathrin-mediated endocytosis), amiloride (macropinocytosis), cytochalasin D (phagocytosis), and filipin complex (caveolae-mediated endocytosis) followed by Perex supplementation is shown. The relative uptake was calculated by the amount of Perex internalized by the cells compared to that in a non-treated control analyzed using flow cytometry. (**D**) Western blot analysis of caveolin-1 protein expression levels. GAPDH was used as an internal control. Full-length blots are also shown in Supplementary Figure S1. All values are expressed as mean ± SD (* *p* < 0.05, ** *p* < 0.01, *** *p* < 0.001, N.S: not significant; *n* = 3).

Perex showed anti-cancer effects only on MDA-MB-231 breast cancer cells; therefore, we explored the reason for this selective effect. One potential reason is that a higher amount of Perex can be taken up by MDA-MB-231 cells than by other cell types. Perex contains components that have anti-cancer properties and delivers them to cells. Thus, a higher uptake rate may contribute to a stronger anticancer effect on cells. To verify whether the selective anti-cancer effect of Perex was due to its uptake capability, the uptake rates of Perex in the seven cell lines were analyzed using flow cytometry. The uptake rates of Perex were 42.3%, 17.0%, 36.1%, 40.7%, 47.8%, 58.2%, and 15.4% for MDA-MB-231, MCF-7, HeLa, A549, SW480, and HEK293T, respectively (Figure 5A,B). Since there were no significant differences in the uptake rates of Perex among several cell lines, including A549, HeLa, SW480, and HEK293T, the selective anti-proliferation and anti-metastasis effects of Perex on MDA-MB-231 cells were not related to the Perex uptake rate.

We hypothesized that the selective anti-cancer effect of Perex among MDA-MB-231, other cancer, and normal cells originated from a difference in the EV penetration through endocytic mechanisms [42]. Endocytosis can be classified into four major mechanisms: phagocytosis, macropinocytosis, clathrin-mediated endocytosis, and caveolae-mediated endocytosis [47,48]. The internalization via caveolae-mediated endocytosis can bypass fusion to lysosomes that contain high amounts of digestive enzymes [49,50,51,52,53]. Therefore, if MDA-MB-231 cells uptake a higher percentage of Perex through caveolae-mediated endocytosis than other uptake mechanisms, we believe that more of the anti-cancer components in Perex could be delivered into the cytosol in the intact form, by avoiding lysosomal degradation. MDA-MB-231 cells were pre-treated with different endocytosis inhibitors before supplementation with Perex to investigate the Perex uptake mechanism. Chlorpromazine, amiloride, cytochalasin D, and the filipin complex inhibited clathrin-mediated endocytosis, macropinocytosis, phagocytosis, and caveolae-mediated endocytosis, respectively [42,54,55,56]. All inhibitors diminished the uptake rate of Perex by MDA-MB-231 cells, but the filipin complex dramatically inhibited caveolae-mediated endocytosis (Figure 5C and Figure S2). The relative uptake of Perex decreased to 59.1, 49.3, 21.7, and 11.2% with chlorpromazine, amiloride, cytochalasin D, and the filipin complex, respectively. The results confirmed that Perex was internalized in MDA-MB-231 cells through various endocytosis pathways, but predominantly via caveolae-mediated endocytosis. 

Caveolin-1, a key player in caveolae-mediated endocytosis, is overexpressed in solid human tumors and plays an important role in cancer progression [57,58]. Thus, we compared caveolin-1 expression levels between cancer and normal cells to determine whether MDA-MB-231 cells express caveolin-1 the most. Western blot results of caveolin-1 in each cell type showed that a much higher amount of caveolin-1 was expressed in MDA-MB-231 cells than in other cells (Figure 5D). For instance, the caveolin-1 expression level in MDA-MB-231 cells was three times higher than in A549 cells and six times higher than in HeLa cells. Thus, high levels of caveolin-1 in MDA-MB-231 cancer cells contributed to the increased level of caveolae-mediated endocytosis for Perex uptake, which in turn resulted in the protection of anticancer components from Perex from lysosomal degradation. 

In particular, Perex has been confirmed to show potential as a treatment for TNBC. MCF-7 cells are ER- and PR-positive, allowing them to be treated with endocrine therapy like tamoxifen. In contrast, MDA-MB-231 cells are classified as TNBC, marked by the absence of ER, PR, and HER2 receptors. Due to the lack of such specific targets, TNBC does not respond to endocrine treatments, leaving chemotherapy as the only standard treatment option for patients. 

Overall, Perex inhibited cancer cell proliferation, migration, and invasion, especially in cancer cells with high levels of caveolin-1. This is very important because Perex can avoid unwanted damage to normal cells, thereby reducing the side-effects compared to other anti-cancer drugs. The following studies are required to demonstrate the anti-TNBC effect of Perex in vivo. Also, it is necessary to check for the side-effects of Perex, including toxicity and immiunogenicity. In this study, we have confirmed that Perex was internalized through caveolae-mediated endocytosis and exhibited anti-cancer effects. These findings suggest the use of caveolin as a target receptor and a new approach for TNBC treatment. Thus, the development of Perex as a novel anticancer drug can be beneficial, especially for the treatment of tumors with high levels of caveolin-1.

## 3. Materials and Methods

### 3.1. Cell Culture

MDA-MB-231 (human breast cancer), SW480 (human colorectal cancer), MCF-7 (human breast carcinoma), and A549 (human lung cancer) HeLa (human cervical cancer) were purchased from the Korean Cell Line Bank (Seoul, Republic of Korea). HEK293T (human embryonic kidney) cells were a kind gift from Prof. S. H. Kwon at Yonsei University, Republic of Korea. MDA-MB-231, SW480, and HEK293T cells were cultured in Dulbecco’s Modified Eagle Medium (Corning Inc., Corning, NY, USA) supplemented with 10% (*v*/*v*) FBS (Gibco, Waltham, NY, USA) and 1% (*v*/*v*) penicillin/streptomycin (Gibco, Waltham, NY, USA). MCF-7 and A549 cells were maintained in RPMI-1640 medium containing 10% FBS and 1% penicillin/streptomycin. HeLa cells were cultured in Minimum Essential Media (Corning Inc., Corning, NY, USA) supplemented with 10% FBS and 1% penicillin/streptomycin. All cells were incubated at 37 °C in a humidified 5% CO_2_ environment.

### 3.2. Preparation and Isolation of Extracellular Vesicles

For the preparation of Perex, perilla leaves were purchased from the local market in Korea, in accordance with the relevant legislation governing the collection and sourcing of plant material. The appropriate permissions were obtained for the acquisition of perilla leaves. The perilla leaves were washed with distilled water to remove dust and pesticides. Perilla leaf juice was prepared using a blender, and large debris was removed through serial centrifugation at 8000× *g* and 20,000× *g* for 1 h. Then, an Amicon Ultra-15 filter unit (Millipore, Burlington, MA, USA) was used to concentrate the perilla leaf juice. The samples were centrifuged at 5000× *g* for 4 h at 4 °C, followed by EV isolation using size-exclusion chromatography (Izon Science, Addington, Christchurch, New Zealand). The fractions were eluted with PBS, and EV and protein concentrations were assessed in each fraction.

### 3.3. Characterization of Extracellular Vesicles

The size distribution and concentration of Perex were assessed using Nano Tracking Analysis (Nanosight, NS300, Malvern Panalytical, Malvern, Worcestershire, UK). The same camera level, threshold, and focus were used for all the assessments. For transmission electron microscopy (TEM) imaging, the sample was applied to carbon-coated copper grids (200 mesh, Electron Microscopy Sciences, Hatfield, PA, USA). After allowing the sample to absorb for 2 min and blotting off the buffer solution onto Whatman paper, the sample on the grids was stained with 2% (*w*/*v*) uranyl acetate for 1 min. Distilled water was then added for 1 min to remove uranyl acetate, followed by drying for 15 min. TEM images were captured using a Bio-High-voltage EM system (120 kV, JEM-1400 Plus, JEOL Ltd., Tokyo, Japan) at the Korea Basic Science Institute. The zeta potential and polydispersity index (PDI) were measured through dynamic light scattering (DLS) analysis using Zetasizer NS (Malvern Panalytical, Malvern, Worcestershire, UK) at 25 °C.

### 3.4. Internalization of Perex into Mammalian Cells

To verify that Perex can be internalized by mammalian cells and compare the rate of internalization into various cells, Perex was pre-labeled with PKH67 (Sigma-Aldrich, St. Louis, MO, USA) for 15 min at room temperature or DiI dye (Thermo Scientific, Waltham, MA, USA) for 20 min at 37 °C. The mixture was ultrafiltered through a 100 kDa filter to remove the free dye. MDA-MB-231 cells were seeded and cultured in a medium with PKH-labeled Perex at a concentration of 1.0 × 10^11^ particles/mL. After incubation, Hoechst 33342 fluorescent dye (Cell Signaling Technology, Danvers, MA, USA) was added to the culture medium for nuclear staining. Cells were washed several times and observed under a fluorescence microscope (Nikon Corp., Tokyo, Japan). Cancer and normal cells were treated with DiI-labeled Perex at a concentration of 1.0 × 10^11^ particles/mL and analyzed using a flow cytometer (Beckman Coulter Inc., Brea, CA, USA).

### 3.5. Measurement of Cell Proliferation

To estimate the anti-cancer effect of Perex, cancer and normal cells were treated with different concentrations of Perex ranging from 1.0 × 10^9^ to 2.0 × 10^11^ particles/mL from 48 to 120 h. Cell proliferation was measured using a WST-1 (2-(4-iodophenyl)-3-(4-nitrophenyl)-5-(2,4-disulfophenyl)-2H-tetrazolium) assay kit (EZ-Cytox, DoGenBio, Seoul, Korea). The reagent was mixed with cell culture media at a ratio of 1:10. And the mixed reagent (100 μL) was added to a 96-well plate and incubated for 1 h at 37 °C. The optical density was measured at 450 nm. 

### 3.6. Cell Migration and Invasion Assays

To assess the anti-metastatic effect of Perex on MDA-MB-231 and A549 cells, a migration assay was performed using Transwell plates (24-well, 8 μm pore size; Corning Inc., Corning, NY, USA). An in vitro invasion assay was performed using Matrigel (Corning Inc., Corning, NY, USA). Transwell inserts were coated with 100 μL of 200 μg/mL Matrigel diluted in coating buffer at 37 °C. MDA-MB-231 and A549 cells were seeded into the upper chamber containing Perex (2 × 10^11^ particles/mL) in serum-free medium. The lower chamber of the Transwell plate was filled with medium containing serum to induce chemotaxis. After incubation for 48 h, the migrated or invaded cells were fixed with 4% paraformaldehyde for 20 min, permeabilized with methanol, and stained with 0.1% crystal violet (Sigma-Aldrich, St. Louis, MO, USA) for 20 min. Non-migrated invaded cells were swabbed with cotton swabs, and Transwell inserts were completely dried before visualization. The migrated and invaded cells were imaged under a light microscope and counted in five random areas in each Transwell insert.

### 3.7. Endocytosis Inhibitor Assay

To explore the endocytic pathway of Perex, MDA-MB-231 cells were pretreated with various endocytosis inhibitors, including 10 μg/mL chlorpromazine (Sigma-Aldrich, St. Louis, MO, USA), 10 μg/mL amiloride (Sigma-Aldrich, St. Louis, MO, USA), 0.5 μg/mL cytochalasin D (Sigma-Aldrich, St. Louis, MO, USA), and 20 μg/mL filipin (Sigma-Aldrich, St. Louis, MO, USA) in the exosome-depleted medium. After 1 h of inhibitor treatment, the cells were supplemented with DiI-labeled Perex (1.0 × 10^11^ particles/mL). DiI-labeled Perex uptake was quantified using a flow cytometer (Beckman Coulter Inc., Brea, CA, USA).

### 3.8. Western Blot Analysis

Western blot analysis was performed to further analyze Caveolin-1 protein expression levels. Protein concentration was measured using the BCA assay. Twenty micrograms of protein was separated through SDS-PAGE. The proteins were then transferred onto a nitrocellulose membrane at 65 V for 2 h. The membranes were blocked with 5% skim milk at room temperature for 2 h, and then the membranes were incubated with Caveolin-1 (Cell Signaling Technology, Danvers, MA, USA) and GAPDH (Cell Signaling Technology, Danvers, MA, USA) antibodies overnight at 4 °C. Both antibodies were titrated to 1/1000 as the supplier’s recommendations. After washing, the membranes were incubated with HRP-conjugated secondary antibodies at room temperature for 1 h. Finally, the protein bands were visualized using ECL Blotting Reagent (Cytiva, Marlborough, MA, USA) and scanned on a ChemiDoc™ XRS System (Bio-Rad, Hercules, CA, USA).

### 3.9. Statistical Analysis

All data were independently analyzed more than three times and are represented as mean ± SD. Statistical analyses were performed using one-way ANOVA, two-way ANOVA, and *t*-tests followed by a multiple comparisons test using GraphPad Prism 7.00 (GraphPad Prism Software Inc., San Diego, CA, USA). Differences were considered statistically significant at * *p* < 0.05, ** *p* < 0.01, *** *p* < 0.001, N.S: not significant.

## 4. Conclusions

The development of therapeutics that inhibit cancer cell proliferation and metastasis is important to improve the survival rate and efficacy of cancer treatment. Many traditional chemotherapies for breast cancer have limitations including cytotoxicity to normal cells and poor biocompatibility. Recent studies have shown that some EVs with anticancer activity have great potential as alternative candidates. However, EV productivity using mammalian cell cultures is a critical issue, especially due to its low EV yield. In the present study, we found that a high amount of Perex could be isolated from perilla leaves. In addition, Perex showed anti-cancer properties and inhibited the proliferation, migration, and invasion of MDA-MB-231 cancer cells that have high levels of caveolin-1 compared with other cancer and normal cells. This contributes to the selective attack of cancer cells with high levels of caveolin-1 and the reduction in unwanted side-effects on normal cells. Considering its high productivity, low production cost, selective anticancer effects, and few side-effects, Perex is a promising candidate for the therapeutic treatment of breast cancer.

## Data Availability

The data presented in this study are available on request from the corresponding author. The data are not publicly available due to privacy.

## Supplementary Materials

The following supporting information can be downloaded at https://www.mdpi.com/article/10.3390/ijms242115633/s1.

## References

[1] Tian F., Zhang S., Liu C., Han Z., Liu Y., Deng J., Li Y., Wu X., Cai L., Qin L. (2021). Protein analysis of extracellular vesicles to monitor and predict therapeutic response in metastatic breast cancer. Nat. Commun..

[2] Wu Z.-H., Tang Y., Yu H., Li H.-D. (2021). The role of ferroptosis in breast cancer patients: A comprehensive analysis. Cell Death Discov..

[3] Jiang K., Song X., Yang L., Li L., Wan Z., Sun X., Gong T., Lin Q., Zhang Z. (2018). Enhanced antitumor and anti-metastasis efficacy against aggressive breast cancer with a fibronectin-targeting liposomal doxorubicin. J. Control. Release.

[4] Medeiros B., Allan A.L. (2019). Molecular Mechanisms of Breast Cancer Metastasis to the Lung: Clinical and Experimental Perspectives. Int. J. Mol. Sci..

[5] Theodossiou T.A., Ali M., Grigalavicius M., Grallert B., Dillard P., Schink K.O., Olsen C.E., Walchli S., Inderberg E.M., Kubin A. (2019). Simultaneous defeat of MCF7 and MDA-MB-231 resistances by a hypericin PDT-tamoxifen hybrid therapy. NPJ Breast Cancer.

[6] Grubczak K., Kretowska-Grunwald A., Groth D., Poplawska I., Eljaszewicz A., Bolkun L., Starosz A., Holl J.M., Mysliwiec M., Kruszewska J. (2021). Differential Response of MDA-MB-231 and MCF-7 Breast Cancer Cells to In Vitro Inhibition with CTLA-4 and PD-1 through Cancer-Immune Cells Modified Interactions. Cells.

[7] Huang K.S., Wang Y.T., Byadgi O., Huang T.Y., Tai M.H., Shaw J.F., Yang C.H. (2022). Screening of Specific and Common Pathways in Breast Cancer Cell Lines MCF-7 and MDA-MB-231 Treated with Chlorophyllides Composites. Molecules.

[8] Lee Y.K., Bae K., Yoo H.S., Cho S.H. (2018). Benefit of Adjuvant Traditional Herbal Medicine with Chemotherapy for Resectable Gastric Cancer. Integr. Cancer Ther..

[9] Pedrosa P., Mendes R., Cabral R., Martins L., Baptista P.V., Fernandes A.R. (2018). Combination of chemotherapy and Au-nanoparticle photothermy in the visible light to tackle doxorubicin resistance in cancer cells. Sci. Rep..

[10] Liu C.T., Chen Y.H., Huang Y.C., Chen S.Y., Tsai M.Y. (2019). Chemotherapy in conjunction with traditional Chinese medicine for survival of patients with early female breast cancer: Protocol for a non-randomized, single center prospective cohort study. Trials.

[11] Hartjes T.A., Mytnyk S., Jenster G.W., van Steijn V., van Royen M.E. (2019). Extracellular Vesicle Quantification and Characterization: Common Methods and Emerging Approaches. Bioengineering.

[12] Walker S., Busatto S., Pham A., Tian M., Suh A., Carson K., Quintero A., Lafrence M., Malik H., Santana M.X. (2019). Extracellular vesicle-based drug delivery systems for cancer treatment. Theranostics.

[13] Doyle L.M., Wang M.Z. (2019). Overview of Extracellular Vesicles, Their Origin, Composition, Purpose, and Methods for Exosome Isolation and Analysis. Cells.

[14] Raghav A., Jeong G.B. (2021). A systematic review on the modifications of extracellular vesicles: A revolutionized tool of nano-biotechnology. J. Nanobiotechnology.

[15] Ramasubramanian L., Kumar P., Wang A. (2019). Engineering extracellular vesicles as nanotherapeutics for regenerative medicine. Biomolecules.

[16] Szatanek R., Baj-Krzyworzeka M., Zimoch J., Lekka M., Siedlar M., Baran J. (2017). The methods of choice for extracellular vesicles (EVs) characterization. Int. J. Mol. Sci..

[17] Jia Y., Chen Y., Wang Q., Jayasinghe U., Luo X., Wei Q., Wang J., Xiong H., Chen C., Xu B. (2017). Exosome: Emerging biomarker in breast cancer. Oncotarget.

[18] Yang H.C., Rhee W.J. (2021). Single step in situ detection of surface protein and microRNA in clustered extracellular vesicles using flow cytometry. J. Clin. Med..

[19] Cho S., Yang H.C., Rhee W.J. (2019). Simultaneous multiplexed detection of exosomal microRNAs and surface proteins for prostate cancer diagnosis. Biosens. Bioelectron..

[20] Jeong K., Yu Y.J., You J.Y., Rhee W.J., Kim J.A. (2020). Exosome-mediated microRNA-497 delivery for anti-cancer therapy in a microfluidic 3D lung cancer model. Lab Chip.

[21] Kim H., Rhee W.J. (2020). Exosome-mediated let7c-5p delivery for breast cancer therapeutic development. Biotechnol. Bioprocess Eng..

[22] Kim H., Kang S.J., Rhee W.J. (2021). Phenylboronic Acid-conjugated Exosomes for Enhanced Anticancer Therapeutic Effect by Increasing Doxorubicin Loading Efficiency. Biotechnol. Bioprocess Eng..

[23] Qiu X., Liu J., Zheng C., Su Y., Bao L., Zhu B., Liu S., Wang L., Wang X., Wang Y. (2020). Exosomes released from educated mesenchymal stem cells accelerate cutaneous wound healing via promoting angiogenesis. Cell Prolif..

[24] Zhang S., Chuah S.J., Lai R.C., Hui J.H.P., Lim S.K., Toh W.S. (2018). MSC exosomes mediate cartilage repair by enhancing proliferation, attenuating apoptosis and modulating immune reactivity. Biomaterials.

[25] Even M.S., Sandusky C.B., Barnard N.D. (2006). Serum-free hybridoma culture: Ethical, scientific and safety considerations. Trends Biotechnol..

[26] Gottipamula S., Muttigi M., Kolkundkar U., Seetharam R. (2013). Serum-free media for the production of human mesenchymal stromal cells: A review. Cell Prolif..

[27] Dad H.A., Gu T.-W., Zhu A.-Q., Huang L.-Q., Peng L.-H. (2021). Plant exosome-like nanovesicles: Emerging therapeutics and drug delivery nanoplatforms. Mol. Ther..

[28] Mu J., Zhuang X., Wang Q., Jiang H., Deng Z.B., Wang B., Zhang L., Kakar S., Jun Y., Miller D. (2014). Interspecies communication between plant and mouse gut host cells through edible plant derived exosome-like nanoparticles. Mol. Nutr. Food Res..

[29] Kim J., Li S., Zhang S., Wang J. (2022). Plant-derived exosome-like nanoparticles and their therapeutic activities. Asian J. Pharm. Sci..

[30] Ju S., Mu J., Dokland T., Zhuang X., Wang Q., Jiang H., Xiang X., Deng Z.-B., Wang B., Zhang L. (2013). Grape exosome-like nanoparticles induce intestinal stem cells and protect mice from DSS-induced colitis. Mol. Ther..

[31] Suharta S., Barlian A., Hidajah A.C., Notobroto H.B., Ana I.D., Indariani S., Wungu T.D.K., Wijaya C.H. (2021). Plant-derived exosome-like nanoparticles: A concise review on its extraction methods, content, bioactivities, and potential as functional food ingredient. J. Food Sci..

[32] Özkan İ., Koçak P., Yıldırım M., Ünsal N., Yılmaz H., Telci D., Şahin F. (2021). Garlic (*Allium sativum*)-derived SEVs inhibit cancer cell proliferation and induce caspase mediated apoptosis. Sci. Rep..

[33] You J.Y., Kang S.J., Rhee W.J. (2021). Isolation of cabbage exosome-like nanovesicles and investigation of their biological activities in human cells. Bioac. Mater..

[34] Yin L., Yan L., Yu Q., Wang J., Liu C., Wang L., Zheng L. (2022). Characterization of the microRNA profile of ginger exosome-like nanoparticles and their anti-inflammatory effects in intestinal Caco-2 cells. J. Agric. Food Chem..

[35] Deng Z., Rong Y., Teng Y., Mu J., Zhuang X., Tseng M., Samykutty A., Zhang L., Yan J., Miller D. (2017). Broccoli-derived nanoparticle inhibits mouse colitis by activating dendritic cell AMP-activated protein kinase. Mol. Ther..

[36] Chen X., Zhou Y., Yu J. (2019). Exosome-like nanoparticles from ginger rhizomes inhibited NLRP3 inflammasome activation. Mol. Pharm..

[37] Yamasaki M., Yamasaki Y., Furusho R., Kimura H., Kamei I., Sonoda H., Ikeda M., Oshima T., Ogawa K., Nishiyama K. (2021). Onion (*Allium cepa* L.)-derived nanoparticles inhibited lps-induced nitrate production, however, their intracellular incorporation by endocytosis was not involved in this effect on RAW264 cells. Molecules.

[38] Perut F., Roncuzzi L., Avnet S., Massa A., Zini N., Sabbadini S., Giampieri F., Mezzetti B., Baldini N. (2021). Strawberry-derived exosome-like nanoparticles prevent oxidative stress in human mesenchymal stromal cells. Biomolecules.

[39] Kim D.K., Rhee W.J. (2021). Antioxidative Effects of Carrot-Derived Nanovesicles in Cardiomyoblast and Neuroblastoma Cells. Pharmaceutics.

[40] Zhang L., He F., Gao L., Cong M., Sun J., Xu J., Wang Y., Hu Y., Asghar S., Hu L. (2021). Engineering exosome-like nanovesicles derived from asparagus cochinchinensis can inhibit the proliferation of hepatocellular carcinoma cells with better safety profile. Int. J. Nanomed..

[41] Yang M., Liu X., Luo Q., Xu L., Chen F. (2020). An efficient method to isolate lemon derived extracellular vesicles for gastric cancer therapy. J. Nanobiotechnology.

[42] Kim K., Yoo H.J., Jung J.-H., Lee R., Hyun J.-K., Park J.-H., Na D., Yeon J.H. (2020). Cytotoxic effects of plant sap-derived extracellular vesicles on various tumor cell types. J. Funct. Biomater..

[43] Sidhom K., Obi P.O., Saleem A. (2020). A review of exosomal isolation methods: Is size exclusion chromatography the best option?. Int. J. Mol. Sci..

[44] Guan S., Yu H., Yan G., Gao M., Sun W., Zhang X. (2020). Characterization of urinary exosomes purified with size exclusion chromatography and ultracentrifugation. J. Proteome Res..

[45] Ng K.S., Smith J.A., McAteer M.P., Mead B.E., Ware J., Jackson F.O., Carter A., Ferreira L., Bure K., Rowley J.A. (2019). Bioprocess decision support tool for scalable manufacture of extracellular vesicles. Biotechnol. Bioeng..

[46] Yang H.C., Ham Y.M., Kim J.A., Rhee W.J. (2021). Single-step equipment-free extracellular vesicle concentration using super absorbent polymer beads. J. Extracell. Vesicles.

[47] Miaczynska M., Stenmark H. (2008). Mechanisms and functions of endocytosis. J. Cell Biol..

[48] El-Sayed A., Harashima H. (2013). Endocytosis of gene delivery vectors: From clathrin-dependent to lipid raft-mediated endocytosis. Mol. Ther..

[49] Song Y., Wu Y., Xu L., Jiang T., Tang C., Yin C. (2021). Caveolae-mediated endocytosis drives robust siRNA delivery of polymeric nanoparticles to macrophages. ACS Nano.

[50] Qiu C., Han H.-H., Sun J., Zhang H.-T., Wei W., Cui S.-H., Chen X., Wang J.-C., Zhang Q. (2019). Regulating intracellular fate of siRNA by endoplasmic reticulum membrane-decorated hybrid nanoplexes. Nat. Commun..

[51] Reilly M.J., Larsen J.D., Sullivan M.O. (2012). Polyplexes traffic through caveolae to the Golgi and endoplasmic reticulum en route to the nucleus. Mol. Pharm..

[52] Zhang L., Yang X., Lv Y., Xin X., Qin C., Han X., Yang L., He W., Yin L. (2017). Cytosolic co-delivery of miRNA-34a and docetaxel with core-shell nanocarriers via caveolae-mediated pathway for the treatment of metastatic breast cancer. Sci. Rep..

[53] Li Y., Wu Z., He W., Qin C., Yao J., Zhou J., Yin L. (2015). Globular protein-coated Paclitaxel nanosuspensions: Interaction mechanism, direct cytosolic delivery, and significant improvement in pharmacokinetics. Mol. Pharm..

[54] Wang X., Hu S., Li J., Zhu D., Wang Z., Cores J., Cheng K., Liu G., Huang K. (2021). Extruded Mesenchymal Stem Cell Nanovesicles Are Equally Potent to Natural Extracellular Vesicles in Cardiac Repair. ACS Appl. Mater. Interfaces.

[55] Sawada S.I., Sato Y.T., Kawasaki R., Yasuoka J.I., Mizuta R., Sasaki Y., Akiyoshi K. (2020). Nanogel hybrid assembly for exosome intracellular delivery: Effects on endocytosis and fusion by exosome surface polymer engineering. Biomater. Sci..

[56] Nam H.Y., Kwon S.M., Chung H., Lee S.Y., Kwon S.H., Jeon H., Kim Y., Park J.H., Kim J., Her S. (2009). Cellular uptake mechanism and intracellular fate of hydrophobically modified glycol chitosan nanoparticles. J. Control. Release.

[57] Tse E.Y., Ko F.C., Tung E.K., Chan L.K., Lee T.K., Ngan E.S., Man K., Wong A.S., Ng I.O., Yam J.W. (2012). Caveolin-1 overexpression is associated with hepatocellular carcinoma tumourigenesis and metastasis. J. Pathol..

[58] Ketteler J., Klein D. (2018). Caveolin-1, cancer and therapy resistance. Int. J. Cancer.

