# Supplementary material for: Perilla-Leaf-Derived Extracellular Vesicles Selectively Inhibit Breast Cancer Cell Proliferation and Invasion
Source: Int J Mol Sci. 2023 Oct 26;24(21):15633. doi: 10.3390/ijms242115633 (PMC10647566; doi:10.3390/ijms242115633)
Supplement: Supplementary file 1 [file ijms-24-15633-s001.zip › ijms-2671461-supplementary.pdf]

Supplementary Information

Perilla leaf-derived extracellular vesicles selectively inhibit breast cancer cell proliferation and invasion

Do Kyung Kim [1], Su Jin Kang [1] and Won Jong Rhee [1,2,3,*]

[1] Department of Bioengineering and Nano-Bioengineering, Incheon National University, Incheon, 22012, Republic of Korea
[2] Division of Bioengineering, Incheon National University, Incheon 22012, Republic of Korea
[3] Research Center for Bio Materials & Process Development, Incheon National University, 119 Academy-ro, Yeonsu-gu, Incheon 22012, Republic of Korea

* Correspondence: wjrhee@inu.ac.kr; Tel.: +82 32 835 8299; Fax: +82 32 835 0763

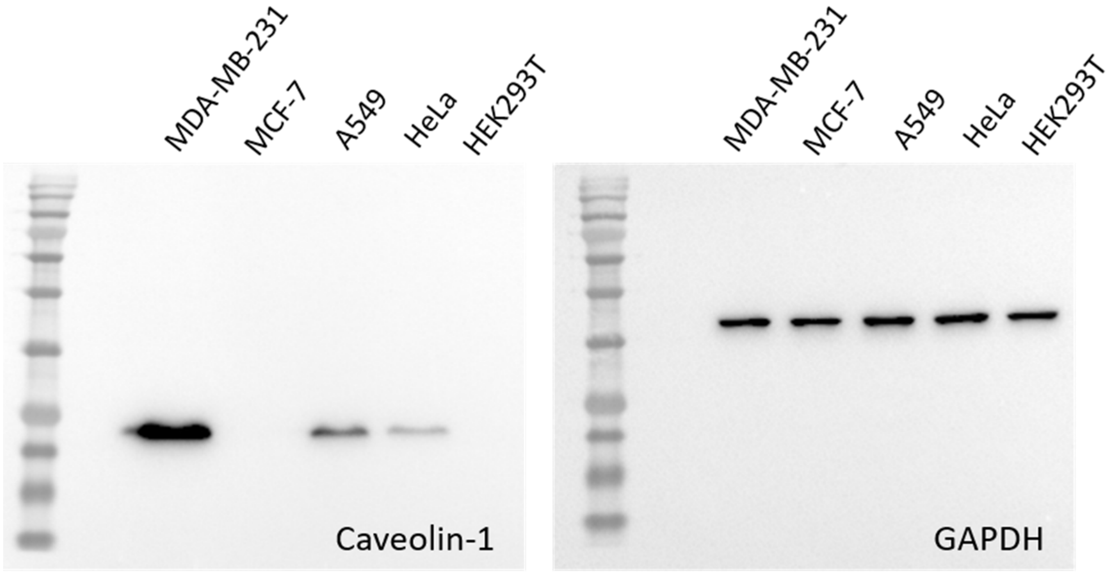

Figure S1. Investigation of Perex uptake mechanism. Full-length western blot results of caveolin-1 and GAPDH are shown.

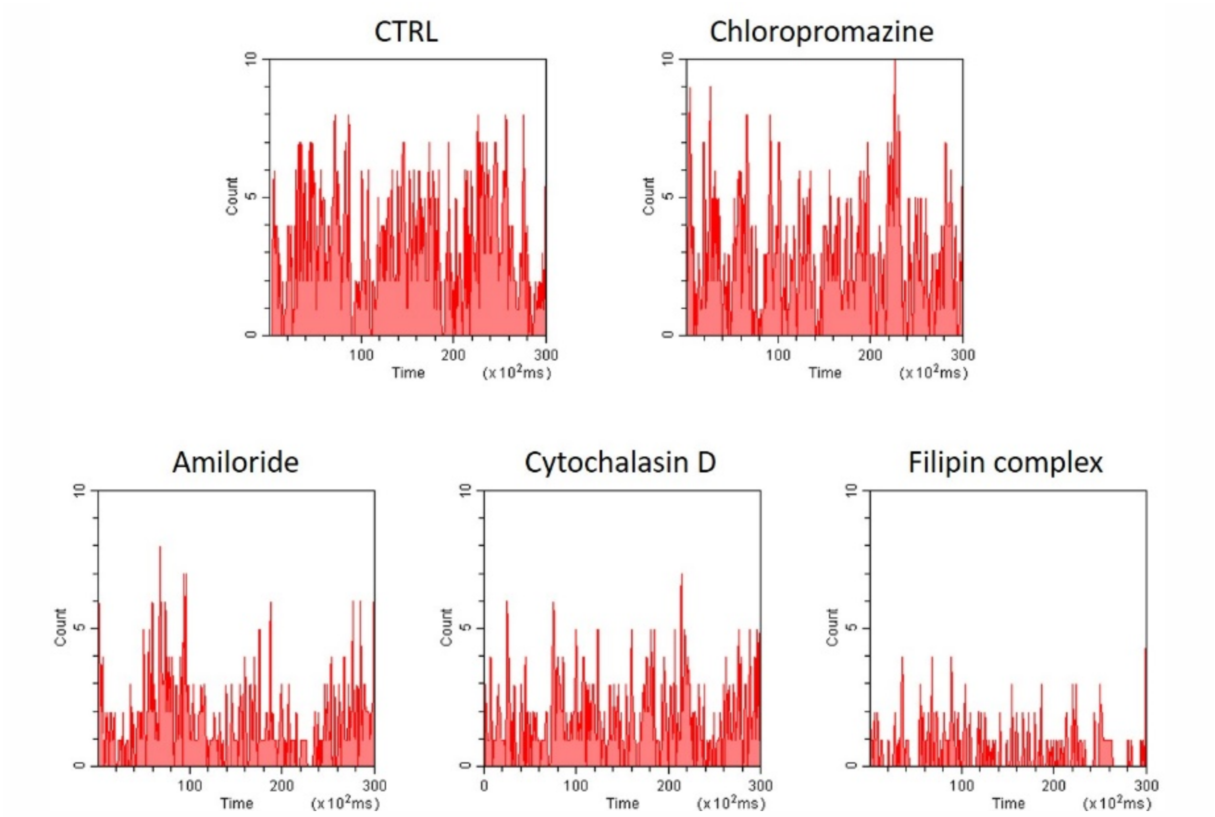

Figure S2. Investigation of Perex uptake mechanism. DiI-labeled Perex was delivered to MDA-MB-231 cells, and the cell numbers that uptook Perex during flow cytometric analysis is measured. Cellular uptake of Perex by MDA-MB-231 cells was compared to those pre-treated with chlorpromazine, amiloride, cytochalasin D, and filipin complex followed by Perex supplementation is shown.
